# Deep learning–based diagnosis of osteoblastic bone metastases and bone islands in computed tomograph images: a multicenter diagnostic study

**DOI:** 10.1007/s00330-023-09573-5

**Published:** 2023-04-15

**Authors:** Yuchao Xiong, Wei Guo, Zhiping Liang, Li Wu, Guoxi Ye, Ying-ying Liang, Chao Wen, Feng Yang, Song Chen, Xu-wen Zeng, Fan Xu

**Affiliations:** 1 Department of Radiology, Guangzhou Red Cross Hospital (Guangzhou Red Cross Hospital, Medical College of Jinan University), 396 Tongfu Road, Guangzhou, 510220 Guangdong Province China; 2 Department of Radiology, Wuhan Third Hospital, Tongren Hospital of Wuhan University, 241 Liuyang Road, Wuhan, 430063 Hubei Province China; 3 Department of Radiology, Guangzhou First People’s Hospital, School of Medicine, South China University of Technology, 1Panfu Road, Guangzhou, 510180 Guangdong Province China

**Keywords:** Osteogenesis, Bone neoplasms, Deep learning, Computed tomography, X-ray

## Abstract

**Objective:**

To develop and validate a deep learning (DL) model based on CT for differentiating bone islands and osteoblastic bone metastases.

**Materials and methods:**

The patients with sclerosing bone lesions (SBLs) were retrospectively included in three hospitals. The images from *site 1* were randomly assigned to the training (70%) and intrinsic verification (10%) datasets for developing the two-dimensional (2D) DL model (single-slice input) and “2.5-dimensional” (2.5D) DL model (three-slice input) and to the internal validation dataset (20%) for evaluating the performance of both models. The diagnostic performance was evaluated using the internal validation set from *site 1* and additional external validation datasets from *site 2* and *site 3*. And statistically analyze the performance of 2D and 2.5D DL models.

**Results:**

In total, 1918 SBLs in 728 patients in *site 1*, 122 SBLs in 71 patients in *site 2*, and 71 SBLs in 47 patients in *site 3* were used to develop and test the 2D and 2.5D DL models. The best performance was obtained using the 2.5D DL model, which achieved an AUC of 0.996 (95% confidence interval [CI], 0.995–0.996), 0.958 (95% CI, 0.958–0.960), and 0.952 (95% CI, 0.951–0.953) and accuracies of 0.950, 0.902, and 0.863 for the internal validation set, the external validation set from *site 2* and *site 3*, respectively.

**Conclusion:**

A DL model based on a three-slice CT image input (2.5D DL model) can improve the prediction of osteoblastic bone metastases, which can facilitate clinical decision-making.

**Key Points:**

*• This study investigated the value of deep learning models in identifying bone islands and osteoblastic bone metastases.*

*• Three-slice CT image input (2.5D DL model) outweighed the 2D model in the classification of sclerosing bone lesions.*

*• The 2.5D deep learning model showed excellent performance using the internal (AUC, 0.996) and two external (AUC, 0.958; AUC, 0.952) validation sets.*

**Supplementary Information:**

The online version contains supplementary material available at 10.1007/s00330-023-09573-5.

## Introduction

Computed tomography (CT) is widely used for detecting, evaluating, and staging malignant tumors, and sclerosing bone lesions are frequently detected on CT images [1, 2]. Distinguishing a sclerosing bone lesion from an osteoblastic bone metastasis or bone island is essential to determine the next diagnostic steps and prepare a treatment strategy. For patients with a non-tumor history, diagnosing bone islands does not require treatment, and diagnosing osteoblastic bone metastases requires the primary lesion to be found to determine a treatment plan. For tumor patients, distinguishing bone islands from osteoblastic bone metastases can change tumor staging and alter treatment options.

To differentiate between bone islands and osteoblastic bone metastases, many methods have improved the diagnosis of sclerosing bone lesions, such as positron emission tomography (PET)/CT [3], dual-energy CT [4], identification of the salt-and-pepper noise sign in magnetic resonance (MR) images [5], CT attenuation measurements [6], and radiomics [7]. However, PET/CT, dual-energy CT, and MR examinations cannot be performed in many remote locations, and when they can be performed, they increase the financial burden of patients. In addition, radiomic methods and CT attenuation measurements rely on the precise delineation of regions of interest (ROIs), which is largely affected by human factors.

In contrast, advanced deep learning (DL) models overcome these problems by using powerful feature learning capabilities [8, 9] and have shown the potential to help humans in various medical fields [10–12]. In addition, unlike radiomic methods, a classification model based on DL does not require precise delineation of tumor boundaries and can automatically learn to classify features in image data and diagnose conditions accordingly. Moreover, external verification does not require accurate delineation of the lesions, increasing the possibility of using the model in various medical institutions.

In this study, we constructed a DL model to differentiate between bone islands and osteoblastic bone metastases. Our approach used an end-to-end pipeline that only required the manual selection of lesion regions in CT images and did not require precise lesion boundary segmentation or human-defined features. The proposed model needed only simple lesion delineation to diagnose sclerosing bone lesions. To evaluate the DL model’s performance, we collected two datasets from two independent hospitals and independently validated the results.

## Materials and methods

### Datasets

This multicenter retrospective study was conducted in three hospitals in China. The institutional review board of the principal investigator’s hospital approved the study and waived the requirement for written informed consent.

**Fig. 1:**
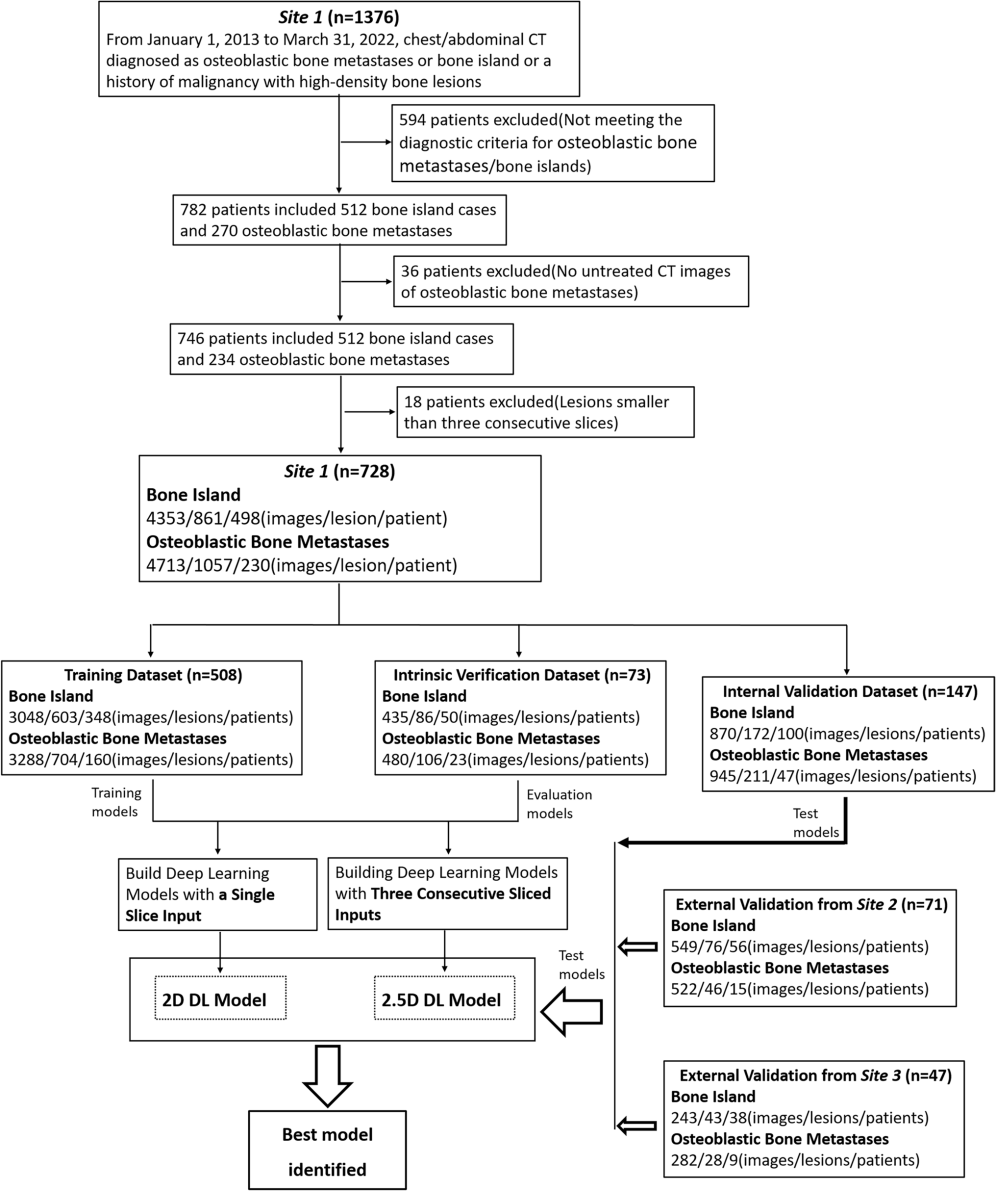
Workflow diagram for the development and evaluation of the 2D DL model and 2.5D DL model to differentiate between bone islands and osteoblastic bone metastases

For the development dataset, we evaluated chest and abdominal CT images acquired at Guangzhou Red Cross Hospital, Guangzhou, China from January 1, 2013, to March 31, 2022, that were reported to contain a bone island or osteoblastic bone metastases and for patients with a history of malignancy with high-density bone lesions. Initially, 1376 patients were recruited. Those who met the following criteria were diagnosed with a bone island: (1) no history of malignancy; (2) lesions denser than trabecular bone that were round or oblong or with speculated margin [6, 13, 14]; (3) no change in size, shape, and density on follow-up CT scans obtained at least 6 months later [7]; and (4) lesions displayed on at least three consecutive CT slices. Patients who met the following criteria were diagnosed with osteoblastic bone metastases: (1) history of malignancy; (2) no bone metastases identified during previous imaging examinations, and local high-density lesions with a maximum diameter of  < 2 cm in the vertebral body or pelvic bone identified in the most recent CT image; (3) lesions displayed on at least three consecutive CT slices; (4) no history of chemotherapy, antiandrogen therapy, or bisphosphonate therapy prior to discovering high-density lesions [6]; and (5) no pathological fractures. Applying the above diagnostic criteria, two experienced radiologists (L.Z.P. and X.F., with 21 and 9 years, respectively, in musculoskeletal radiology) excluded 648 patients, leaving 728 (498 cases of bone islands and 230 cases of osteoblastic bone metastases) enrolled in this study (Fig. 1).

To verify the DL model’s utility in clinical practice, two external validation datasets containing CT images of bone islands and osteoblastic bone metastases acquired between January 1, 2019, and December 31, 2021, were also obtained from Guangzhou Cancer Hospital (GZCH), Guangzhou, China and Wuhan Third Hospital (WHTH), Wuhan, China.

### Image reading and annotation

**Fig. 2:**
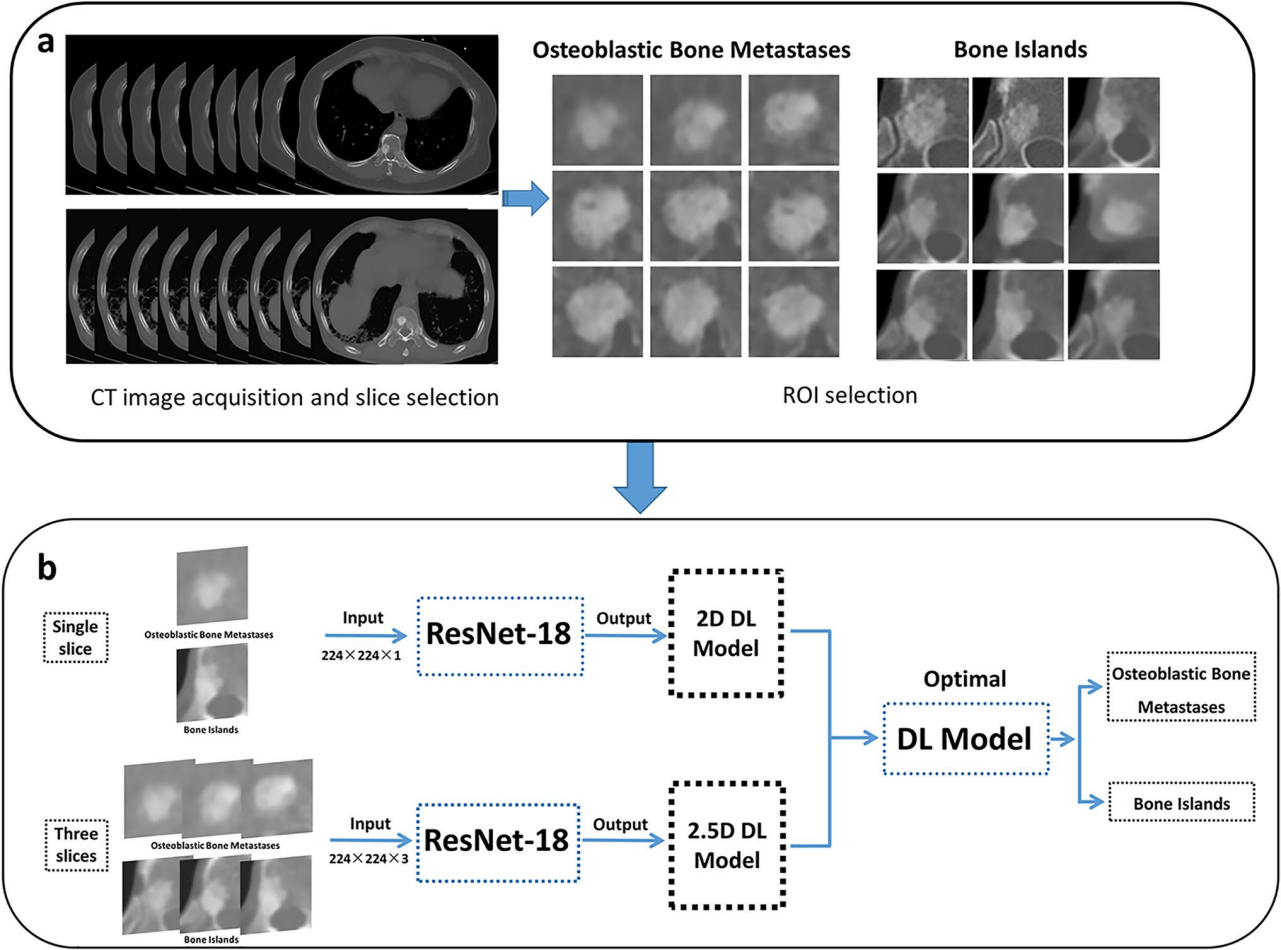
Flow chart for building a deep learning model based on CT images. **a** CT image acquisition and segmentation. **b** Residual network and CT-based model construction

According to the diagnosis, images were labeled as bone islands or osteoblastic bone metastases. All CT images were preprocessed on a bone window (window level, 400 HU; window width, 2000 HU). The lesion ROI was manually assigned layer-by-layer with bounding boxes on the CT images from three hospitals by two experienced radiologists (L.Z.P. and X.F.) using the LabelImg software (https://pypi.org/project/labelImg/) and annotated. To apply the DL model, ROIs containing the lesion were manually selected according to the following rules: (1) the ROI of each layer should include the complete lesion area and lesion’s margin; and (2) the number of layers chosen to delineate the lesion should be a multiple of three (Fig. 2a). The rule was implemented easily in practice because it allowed some layers of the lesion to be discarded and the lesion did not need to be exactly in the center of the ROI.

### Development of the DL model

All analyses were performed using Python-based programs. Transfer learning was applied using the ResNet-18 DL model as the basic architecture of the convolutional neural network (CNN). The images from Guangzhou Red Cross Hospital were randomly assigned to the training (70%) and intrinsic verification (10%) datasets for developing the DL model and to the internal validation dataset (20%) for evaluating the DL model’s performance. The data input to the network (Supplementary Figure S1) was divided into a single-slice input (224 × 224 × 1 voxels) and a three-slice input (224 × 224 × 3 voxels) to build two-dimensional (2D) and “2.5-dimensional” (2.5D) DL models, respectively (Fig. 2b). Details of the data preprocessing and the model development are described in the Supplementary Material. The code used for training is stored in GitHub (https://github.com/Xiongyuchao/OBMORBINet).

### Validation of the DL model

The trained 2D and 2.5D DL models were applied to the internal and external test sets. The overall workflow of the 2D and 2.5D DL model development is displayed in Fig. 1.

### Statistical analysis

We used receiver operating characteristic (ROC) curves to demonstrate the ability of DL algorithms to classify sclerosing bone lesions. A ROC curve is generated by plotting the ratio of true positive (TP) cases (sensitivity) to false positive (FP) cases (specificity) by varying the predicted probability threshold. A larger area under the ROC curve (AUC) indicates better diagnostic performance. The code used for data analysis is stored in GitHub (https://github.com/XiongyuchaoOBMORBINet).

## Results

### Clinical characteristics

Clinical characteristics of the development and external test sets are summarized in Table 1. The development set included 9066 images of 1918 sclerosing bone lesions in 498 patients with bone islands and 230 patients with osteoblastic bone metastases. The WHTH external test set included 1071 images of 122 sclerosing bone lesions in 56 patients with bone islands and 15 patients with osteoblastic bone metastases. The GZCH external test set included 525 images of 71 sclerosing bone lesions in 38 patients with bone islands and 9 patients with osteoblastic bone metastases. Detailed patient and lesion information is provided in the Supplementary Material.

**Table 1:** Baseline characteristics

	*Site 1*				External validation		*p*
	Total	Training	Verification	Internal	*Site 2*	*Site 3*	
Number of images	9066	6336	915	1815	1071	525	
Number of lesions	1918	1343	192	383	122	71	
Number of patients	728	508	73	147	71	47	
Age	69.49 ± 13.80	70.22 ± 12.79	68.78 ± 13.93	67.31 ± 16.66	69.65 ± 15.31	65.45 ± 17.29	0.182
Sex							0.073
Man	372	252	37	83	34	32	
Women	356	266	36	64	37	15	
Sclerosing bone lesions							
Bone islands							
Patients	498	348	50	100	56	38	
Lesions	861	603	86	172	76	43	
Images	4353	3048	435	870	549	243	
Osteoblastic bone metastases							
Patients	230	160	23	47	15	9	
Lesions	1057	740	106	211	46	28	
Images	4713	3288	480	945	522	282	

### Establishment of the DL model

After 19 epochs, the training procedure was ended, with no further improvement in accuracy and cross-entropy loss on training and verification for 2D and 2.5D DL models. Using the 2D DL model, an accuracy of up to 98.3% was observed for the training set and 96.1% for the intrinsic verification set. Using the 2.5D DL model, an accuracy of up to 99.7% was observed for the training set and 98.7% for the intrinsic verification set.

### Performance of the 2D and 2.5D DL models

**Fig. 3:**
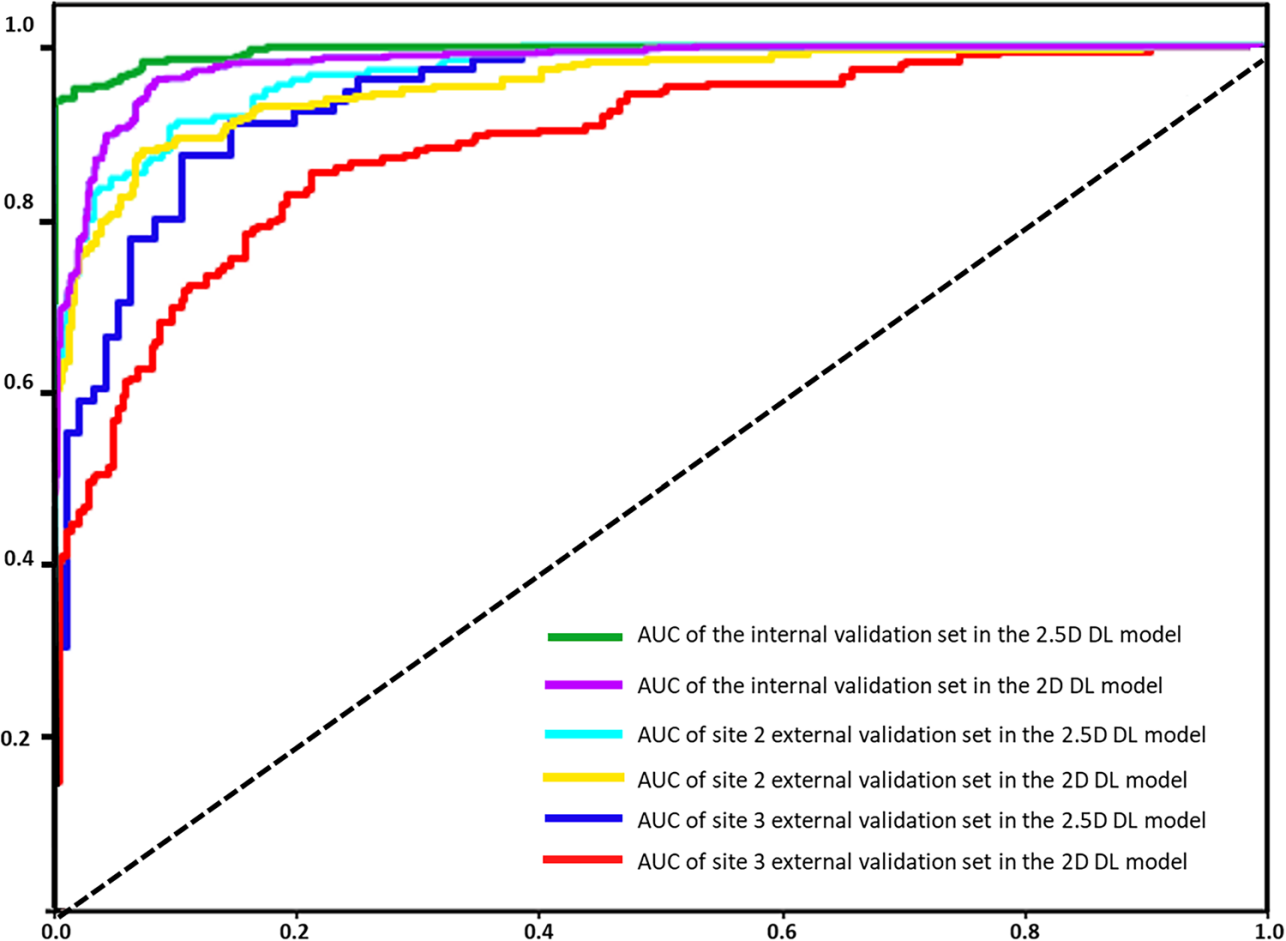
Receiver operating characteristic curves of 2D DL model and 2.5D DL model for differentiating between bone islands and osteoblastic bone metastases in the internal validation set, in the external validation set from site 2, and in the external validation set from site 3

**Fig. 4:**
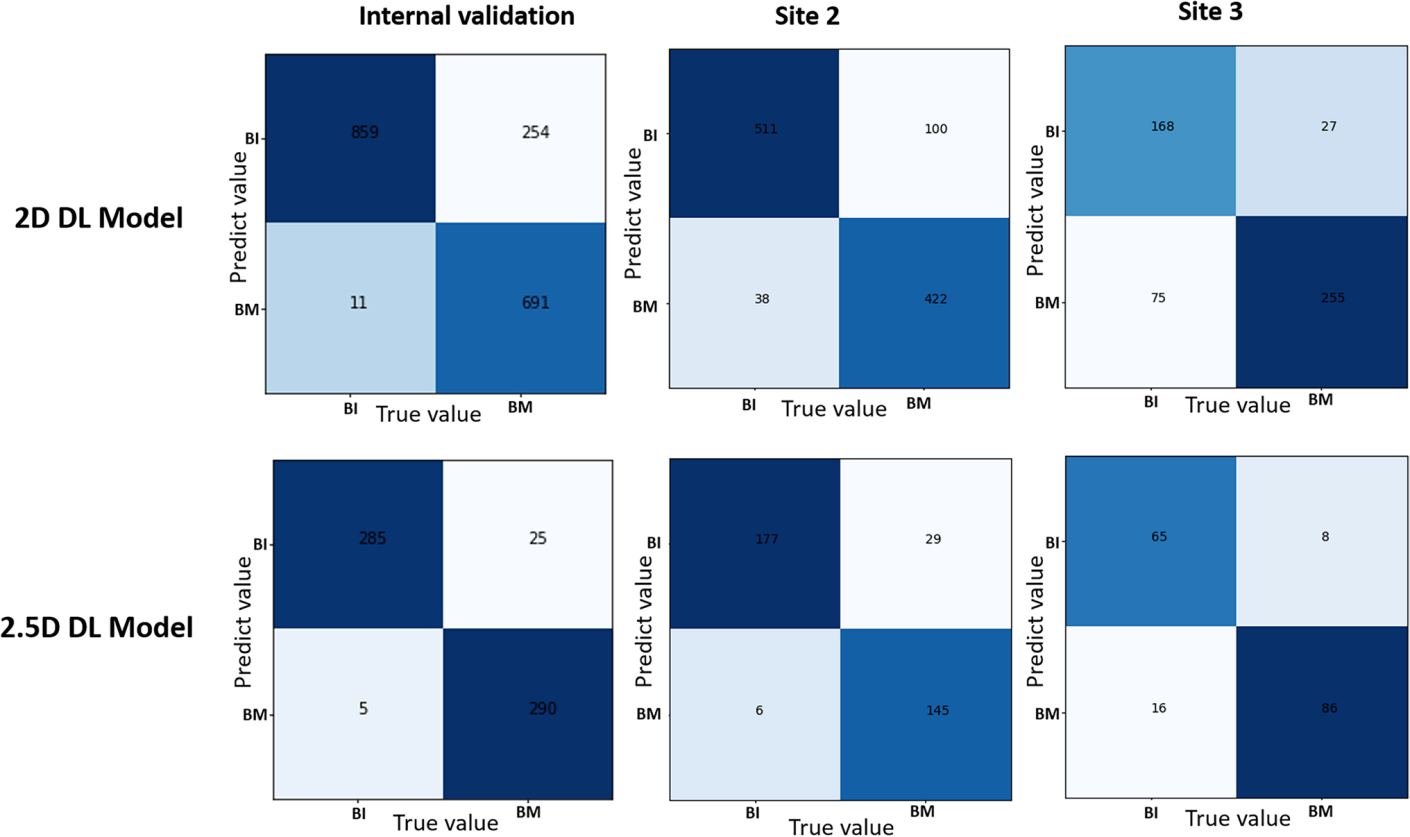
Confusion matrices of the 2D DL model and 2.5D DL model in the internal validation set, in the external validation set from site 2, and in the external validation set from site 3

Both 2D and 2.5D DL models accurately distinguished sclerosing bone lesions in all three validation datasets. The accuracies for the internal validation dataset, external validation dataset from WHTH, and external validation dataset from GZCH were 0.854, 0.871, and 0.806, respectively, for the 2D DL model and 0.950, 0.902, and 0.863, respectively, for the 2.5D DL model (Table 2). Similarly, high AUC values were observed for all three validation datasets, and the AUC of the 2.5D DL model was higher than that of the 2D DL model (Fig. 3). The AUC values for the internal validation dataset, external validation dataset from WHTH, and external validation dataset from GZCH were 0.981(95% CI, 0.980–0.981), 0.940 (95% CI, 0.940–0.941), and 0.890 (95% CI, 0.890–0.892), respectively, for the 2D DL model and 0.996 (95% CI, 0.995–0.996), 0.958 (95% CI, 0.958–0.960), and 0.952 (95% CI, 0.951–0.953), respectively, for the 2.5D DL model. Figure 4 shows confusion matrices for the internal validation dataset, external validation dataset from WHTH, and external validation dataset from GZCH.

**Table 2:** Performance of the 2D DL model and the 2.5D DL model in different validation sets

	Internal validation set		*Site 2*		*Site 3*	
	2D DL model (*n* = 1815)	2.5D DL model (*n* = 605)	2D DL model (*n* = 1071)	2.5D DL model (*n* = 357)	2D DL model (*n* = 525)	2.5D DL model (*n* = 175)
Accuracy	0.854	0.950	0.871	0.902	0.806	0.863
Sensitivity	0.731	0.921	0.808	0.833	0.904	0.915
Specificity	0.987	0.983	0.931	0.967	0.691	0.802
PPV	0.984	0.983	0.917	0.960	0.773	0.843
NPV	0.772	0.919	0.836	0.859	0.862	0.890
F1 Score	0.839	0.951	0.859	0.892	0.833	0.878
FP	11 (0.61%)	5 (0.83%)	38 (3.55%)	6 (1.68%)	75 (14.29%)	16 (9.14%)
FN	254 (14.0%)	25 (4.13%)	100 (9.34%)	29 (8.12%)	27 (5.14%)	8 (4.57%)

*2D DL model*, a deep learning model based on a single slice input; *2.5D DL model*, a deep learning model based on three-slice input

Using the 2.5D DL model, the positive predictive values (PPVs) for the internal validation dataset, external validation dataset from WHTH, and external validation dataset from GZCH were 0.983, 0.960, and 0.843, respectively. The proportion of false negative (FN) detections was less than 4.13% for all validation datasets, 0.83% for the internal validation dataset, 8.12% for the external validation dataset from WHTH, and 4.57% for the external validation dataset from GZCH (Table 2).

**Fig. 5:**
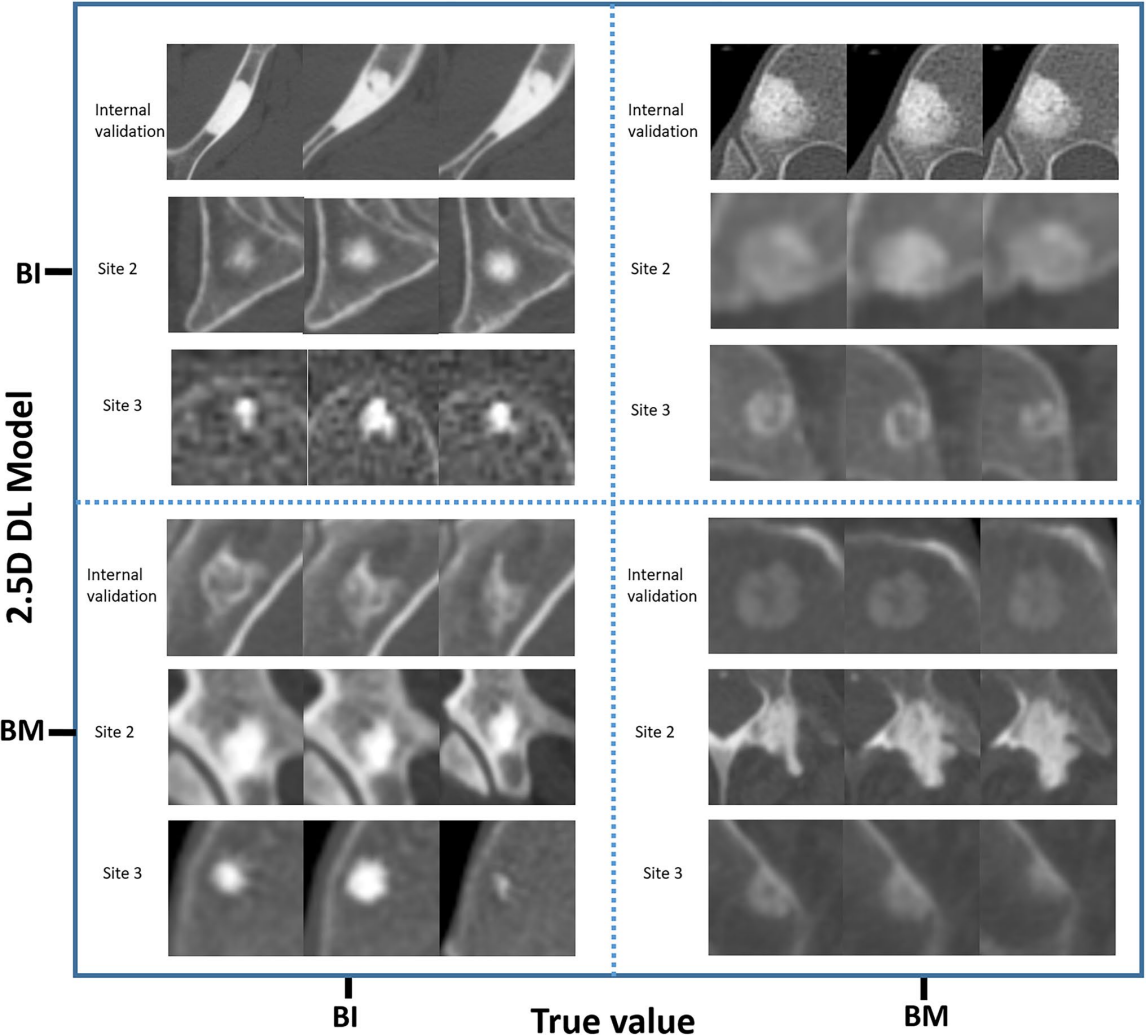
Examples of the 2.5D DL model evaluating correct and incorrect CT images in the internal validation set, in the external validation set from site 2, and in the external validation set from site 3

Using the 2.5D DL model (Fig. 5), FN detections were obtained for 62 three-slice data from 36 lesions, including 25 three-slice data from 14 lesions in the internal validation dataset, 29 three-slice data from 16 lesions in the external validation dataset from WHTH, and 8 three-slice data from 6 lesions in the external validation dataset from GZCH. Of the 36 lesions with FN detections, 25 contained errors in all three-slice data, 11 contained errors in some three-slice data, and 26 of these lesions (41 three-slice data) were adjacent to cortical bone (Supplementary Table S1).

Using the 2.5D DL model, FP detections were obtained for 27 three-slice data from 18 lesions, including 5 three-slice data from 3 lesions in the internal validation dataset, six three-slice data from 3 lesions in the external validation dataset from WHTH, and 16 three-slice data from 12 lesions in the external validation dataset from GZCH. Of the 18 lesions with FP detections, 8 contained errors in all three-slice data, 10 contained errors in some three-slice data, and 7 were adjacent to cortical bone (Supplementary Table S2).

## Discussion

In this study, we separately developed DL models with a single-slice image input (2D DL model) and a continuous three-slice image input (2.5D DL model) to characterize sclerosing bone lesions detected by radiologists using CT. The 2.5D DL model was better able to differentiate between bone islands and osteoblastic bone metastases, with AUC values of 0.996, 0.958, and 0.952 for the internal validation dataset, external validation dataset from WHTH, and external validation dataset from GZCH, respectively; the corresponding values for the 2D DL model were 0.981, 0.940, and 0.890, respectively. The 2.5D DL model differentiated between bone islands and osteoblastic bone metastases in the internal and external validation datasets with high accuracy, sensitivity, and specificity. To the best of our knowledge, this is the first multicenter study to differentiate sclerosing bone lesions using DL.

Although CT has greatly helped to directly distinguish between bone islands and osteoblastic bone metastases using features such as thorny radiation [15], periosteal reaction, soft tissue involvement, and bone destruction [16], only large sclerosing bone lesions can be analyzed and sclerosing bone lesions are often not accompanied by such recognizable imaging features. CT attenuation measurements [6, 13, 17] have shown promising potential to differentiate between bone islands and osteoblastic bone metastases, but their clinical applicability has been jeopardized because CT values are affected by factors such as the region selected for measurement, patient size [18], CT acquisition parameters, and image reconstruction algorithm parameters [19, 20]. CT temporal subtraction is capable of distinguishing osteoblastic bone metastases from bone islands, but its utility is significantly limited for patients with bone islands and those who have osteoblastic bone metastases but received no follow-up CT scan to minimize radiation exposure and/or cost [21, 22]. Several studies [3–5] have explored the potential value of further imaging for diagnosing bone islands and osteoblastic bone metastases. Spectral CT is helpful for distinguishing osteoblastic bone metastases from bone islands, particularly when using the standard deviation of CT values on high-energy virtual monochromatic spectral images [4]. The salt-and-pepper noise sign in bone islands on chemical-shift-encoded MR images can help to differentiate between bone islands and osteoblastic bone metastases [5]. PET/CT helps to differentiate sclerosing bone lesions by assessing tracer uptake [3]. Although the aforementioned studies offer many means of distinguishing between bone islands and osteoblastic bone metastases, they require human visual assessment based on expertise and experience, which is operator-dependent and imposes many demands on imaging equipment that cannot be met in remote areas.

Based on the above, there is currently no objective, simple, and low-cost method to differentiate between osteoblastic bone metastases and bone islands. In current clinical practice, images of patients with no history of tumors and sclerosing bone lesions show typical bone islands that are preferentially diagnosed, but atypical manifestations of bone islands may raise a concern about bone metastases. Patients with a history of tumors, especially those associated with prostate cancer and breast cancer, may be concerned about the possibility of osteoblastic bone metastases even when bone islands appear normal. Osteoblastic bone metastases may present with a bone island-like appearance and thus be initially misdiagnosed, which may delay the treatment of osteoblast bone metastases in tumor patients. There is an urgent need for objective, simple, and low-cost methods to differentiate between osteoblastic bone metastases and bone islands to avoid excessive medical examination of patients with bone islands and delayed treatment of patients with osteoblastic bone metastases.

DL exploits large datasets by directly learning the correlation between complex structures in raw input data and target outputs [23]. Numerous studies have reported the applicability of DL to tumor diagnosis in radiology [24]. However, many that evaluate well-functioning DL models carry a large risk of bias [25]. Using an external cohort is an important validation step to test for bias in DL systems [26]. In this study, the 2.5D DL model yielded a favorable AUC, sensitivity, and specificity for the internal and external test datasets, which indicated that the model could correctly distinguish between bone islands and osteoblastic bone metastases and showed very good generalizability. Hong et al [7] found that CT-based radiomics was helpful for distinguishing between bone islands and osteoblastic bone metastases and yielded better diagnostic performance compared with inexperienced radiologists. Radiomics is a traditional machine learning method that uses carefully chosen representations of input data to predict target outputs. Modern DL techniques are based on CNNs, which use highly flexible artificial neural networks to directly correlate input data to target outputs, and the relationships learned through this correlation are often true [23, 26, 27]. In our study, several advantages of the 2.5D DL model can be highlighted compared with radiomics for distinguishing bone islands from osteoblastic bone metastases. First, the ROI selection based on CNN-based DL only needs to include the complete lesion instead of accurately delineating the lesion edge layer by layer, as in radiomics. This advantage also makes the model more likely to be widely used in other hospitals at all levels. Second, our study recruited more patients, and a larger sample size could obviously ensure better reliability of the classification model, as confirmed by the performance of the 2.5D DL model using the internal and external test datasets. Third, CNN-based DL uses straightforward end-to-end problem-solving that removes the limitations of hand-crafted radiomics features.

The accurate classification of bone islands and osteoblastic bone metastases is of great significance for China, which has a large population, huge differences among the levels of medical resources, and extremely minimal medical insurance funds. Accurate diagnosis of sclerosing bone lesions as bone islands could reduce patient anxiety and the burden on the medical system by avoiding biopsies of benign lesions and unnecessary further imaging studies. The diagnosis of sclerosing bone lesions as osteoblastic bone metastases could allow patients to receive appropriate treatment earlier. However, in current clinical practice, radiologists with different subspecialties have different subjective interpretations of sclerosing bone lesions. Our 2.5D DL model can accurately classify bone islands and osteoblastic bone metastases by simply delineating lesions, which further emphasizes its great clinical value and social benefits. Noguchi et al developed a DL model to identify bone metastases on CT images and this model achieved excellent performance [28]. However, its sensitivity and PPV for osteoblastic bone metastases were significantly lower than those achieved using our 2.5D DL model. Nevertheless, our model can be well combined with that of Noguchi et al to improve the accuracy of osteoblastic bone metastases, which further highlights the clinical value of our study.

In clinical practice, we are more concerned with FN detections produced by the 2.5D DL model. These may result from an ROI that includes adjacent cortical bone. Therefore, we suggest that when radiologists use the 2.5D DL model for osteoblastic bone lesion identification, they should be cautious when lesions are adjacent to cortical bone. Given the urgency of treating osteoblastic bone metastases, the necessary follow-up can be considered cost-effective.

Despite the remarkable results, our study also has some potential limitations. First, the features used by the DL model for classification are difficult to interpret. Therefore, when the doctor’s judgment differs from that of the trained model, the difference cannot be resolved by discussion. Second, this study was retrospective and may suffer from selection bias. Bone islands may be underreported, and many radiologists may not comment on this type of lesion in their reports. Third, only lesions displayed in consecutive slices of three-slice CT images were included in this study. Fourth, this study ignored bone metastases in several locations other than the vertebral body or pelvic bone, the inclusion of which is the goal of our future research.

In conclusion, we developed a classification model of bone islands and osteoblastic bone metastases that achieved high diagnostic accuracy across different hospitals. The 2.5D DL model demonstrated high accuracy, sensitivity, and specificity in differentiating between bone islands and osteoblastic bone metastases in the internal and external validation datasets. Our proposed DL model is simpler and more accurate than other radiomic models and can be generalized.

## Supplementary Information

Below is the link to the electronic supplementary material.

Supplementary file1 (PDF 482 KB)

## Abbreviations

AUC: Area under the ROC curve
CI: Confidence interval
CNN: Convolutional neural network
CT: Computed tomography
DL: Deep learning
MRI: Magnetic resonance imaging
PACS: Picture archiving and communication systems
PET: Positron emission tomography
ROC: Receiver operating characteristic
ROIs: Lesion regions of interest

## Declarations

### Guarantor

The scientific guarantor of this publication is Fan Xu.

### Statistics and biometry

No complex statistical methods were necessary for this paper.

### Methodology

• retrospective

• diagnostic or prognostic study

• multicenter study
