# Supplementary material for: Deep learning–based diagnosis of osteoblastic bone metastases and bone islands in computed tomograph images: a multicenter diagnostic study
Source: Eur Radiol. 2023 Apr 15;33(9):6359–68. doi: 10.1007/s00330-023-09573-5 (PMC10415522; doi:10.1007/s00330-023-09573-5)
Supplement: Supplementary file 1 — Supplementary file1 (PDF 482 KB) [file 330_2023_9573_MOESM1_ESM.pdf]

Supplementary Material

1. Development of the DL Model
2. Detailed patients and lesions information
3. Table S1: False Negative Data Details in the 2.5D DL Model
4. Table S2: False Negative Data Details in the 2.5D DL Model
5. Table S3: The CT protocol of the three independent centers
6. Figure S1. The process of generating input images to the 2.5D DL model

Development of the DL Model

During pre-training, we trialed VGG-19, InceptionV2, Resnet-18, and Resnet-50 models and concluded that Resnet-18 model was the most suitable for this research.

The pre-trained ResNet-18 was performed on Imagenet, a dataset with millions of pictures, and a mean = [0.485, 0.456, 0.406] and standard deviation = [0.229, 0.224, 0.225] is the mean and standard deviation of the Imagenet dataset. We used this mean and standard deviation to normalize the images obtained at our hospital, and using the same value for normalized pre-processing is more conducive to the convergence of the transfer learning model.

The input image was resized to 224 × 224 pixels and normalized with mean = [0.485, 0.456, 0.406] and standard deviation = [0.229, 0.224, 0.225].The 2D DL model superimposes three identical 224 × 224 × 1 images into a 224 × 224 × 3 dataset, and the 2.5D DL model superimposes three different 224 × 224 × 1 images into a 224 × 224 × 3 dataset. These datasets are suitable for use with the pre-trained ResNet-18 model. For training purposes, the training set image was enhanced using horizontal flips, vertical flips, and rotations at random angles. During the training session, the following hyperparameters were used: epochs, 300; mini-batch size, 128; learning rate, 0.0002.

Detailed patients and lesions information.

In the development set, of the 498 patients with bone islands, 247 exhibited synchronous multiple eligible bone islands; 167 had two bone islands, 65 had three bone islands, and 15 had more than four bone islands. Of the 230 patients with osteoblastic bone metastases, 220 exhibited synchronous multiple eligible lesions; 26 had two lesions, 116 had three to five lesions, and 78 had more than five lesions. All 230 patients with osteoblastic bone metastases had primary tumors in the prostate (n = 54), lung (n = 100), breast (n = 48), gastric system (n = 9), nasopharyngeal region (n = 2), kidney (n = 2), pancreas (n = 4), and colon (n = 11).

In the WHTH external test set, of the 56 patients with bone islands, 16 exhibited synchronous multiple eligible bone islands: 12 had two bone islands and 4 had three bone islands. Of the 15 patients with osteoblastic bone metastases, 13 exhibited synchronous multiple eligible lesions: 4 had two lesions, 7 had three to five lesions, and 2 had more than five lesions. All 15 patients with osteoblastic bone metastases had primary tumors in the prostate (n = 2), lung (n = 8), and breast (n

= 5).

In the GZCH external test set, of the 38 patients with bone islands, 4 exhibited synchronous multiple eligible bone islands: 3 had two bone islands and 1 had three bone islands. Of the nine patients with osteoblastic bone metastases, eight exhibited synchronous multiple eligible lesions: two had two lesions, four had two to five lesions, and two had more than five lesions. All nine patients with osteoblastic bone metastases had primary tumors, in the prostate (n = 1), lung (n = 6), and breast (n = 2).

Table S1:False Negative Data Details in the 2.5D DL Model

	Osteoblastic Bone Metastases	Total (Number of three-slice inputs)	Ture (Number of three-slice inputs)	False (Number of three-slice inputs)	Lesion adjacent to cortical bone （YES/NO）
**Internal validation Set**	Lesion 1	5	4	**1**	YES
	Lesion 2	4	0	**4**	YES
	Lesion 3	3	2	**1**	YES
	Lesion 4	3	2	**1**	YES
	Lesion 5	1	0	**1**	YES
	Lesion 6	1	0	**1**	YES
	Lesion 7	2	0	**1**	YES
	Lesion 8	3	0	**3**	YES
	Lesion 9	1	0	**1**	YES
	Lesion 10	2	0	**2**	YES
	Lesion 11	3	2	**1**	YES
	Lesion 12	2	1	**1**	YES
	Lesion 13	3	0	**3**	YES
	Lesion 14	4	0	**4**	NO
**WHTH**	Lesion 1	2	0	**2**	NO
	Lesion 2	5	4	**1**	YES
	Lesion 3	2	0	**2**	YES
	Lesion 4	2	0	**2**	NO
	Lesion 5	2	0	**2**	YES
	Lesion 6	5	4	**1**	YES
	Lesion 7	6	5	**1**	YES
	Lesion 8	5	4	**1**	YES
	Lesion 9	3	0	**3**	YES
	Lesion 10	2	0	**2**	NO
	Lesion 11	2	0	**2**	YES
	Lesion 12	6	5	**1**	YES
	Lesion 13	3	0	**3**	NO
	Lesion 14	3	0	**3**	NO
	Lesion 15	2	0	**2**	YES
	Lesion 16	3	2	**1**	YES
**GZCH**	Lesion 1	1	0	**1**	NO
	Lesion 2	1	0	**1**	NO
	Lesion 3	1	0	**1**	YES
	Lesion 4	2	0	**2**	NO

Lesion 5	1	0	**1**	NO
Lesion 6	2	0	**2**	YES

Table S2: False Positive Data Details in the 2.5D DL Model

	**Bone Islands**	**Total** (Number of three-slice inputs)	**Ture** (Number of three-slice inputs)	**False** (Number of three-slice inputs)	**Lesion adjacent to cortical bone**（YES/NO）
**Internal validation Set**	Lesion 1	1	0	1	YES
	Lesion 2	6	3	3	NO
	Lesion 3	1	0	1	NO
**WHTH**	lesion 1	7	5	2	YES
	lesion 2	6	4	2	NO
	lesion 3	4	2	2	YES
**GZCH**	Lesion 1	1	0	1	YES
	Lesion 2	2	0	2	NO
	Lesion 3	3	2	1	NO
	Lesion 4	1	0	1	NO
	Lesion 5	3	2	1	NO
	Lesion 6	3	2	1	YES
	Lesion 7	2	0	2	NO
	Lesion 8	2	0	2	NO
	Lesion 9	1	0	1	NO
	Lesion 10	4	2	2	YES
	Lesion 11	3	2	1	YES
	Lesion 12	2	1	1	NO

Table S3: The CT protocol of the three independent centers.

Parameters	GZRCH Center		WHTH Center		GZCH Center	
	Chest CT	Abdomen CT	Chest CT	Abdomen CT	Chest CT	Abdomen CT
**CT version**	Philips Brilliance 64	Philips Brilliance 64	UCT 760	UCT 760	Light speed 64 (GE Medical Systems)	Light speed 64 (GE Medical Systems)
**CT tube voltage**	120 kVp	120 kVp	120 kVp	120 kVp	120kvp	120kvp
**CT tube current**	200 mAs	250ms	130 mAs	180 mAs	160mAs	200mAs
**CT rotation time**	0.5s	0.75s	0.5s	0.5s	0.6s	0.6s
**CT detector collimation**	64X0.625	64X0.625	128X0.625	128X0.625	64X0.625	64X0.625
**Image matrix**	512*512	512*512	512*512	512*512	512*512	512*512
**Field of view**	350*350	350*350	500*500	500*500	400*400	400*400
**Section thickness**	3.0mm	3.0mm	1.0mm	1.0mm	1.5mm	1.5mm
**Slice interval**	1.5mm	1.5mm	1.0mm	1.0mm	1.5mm	1.5mm

GZRCH Center, Guangzhou Red Cross Hospital; WHTH Center, Wuhan Third Hospital; GZCH Center, Guangzhou Cancer Hospital; CT, Computed tomography.

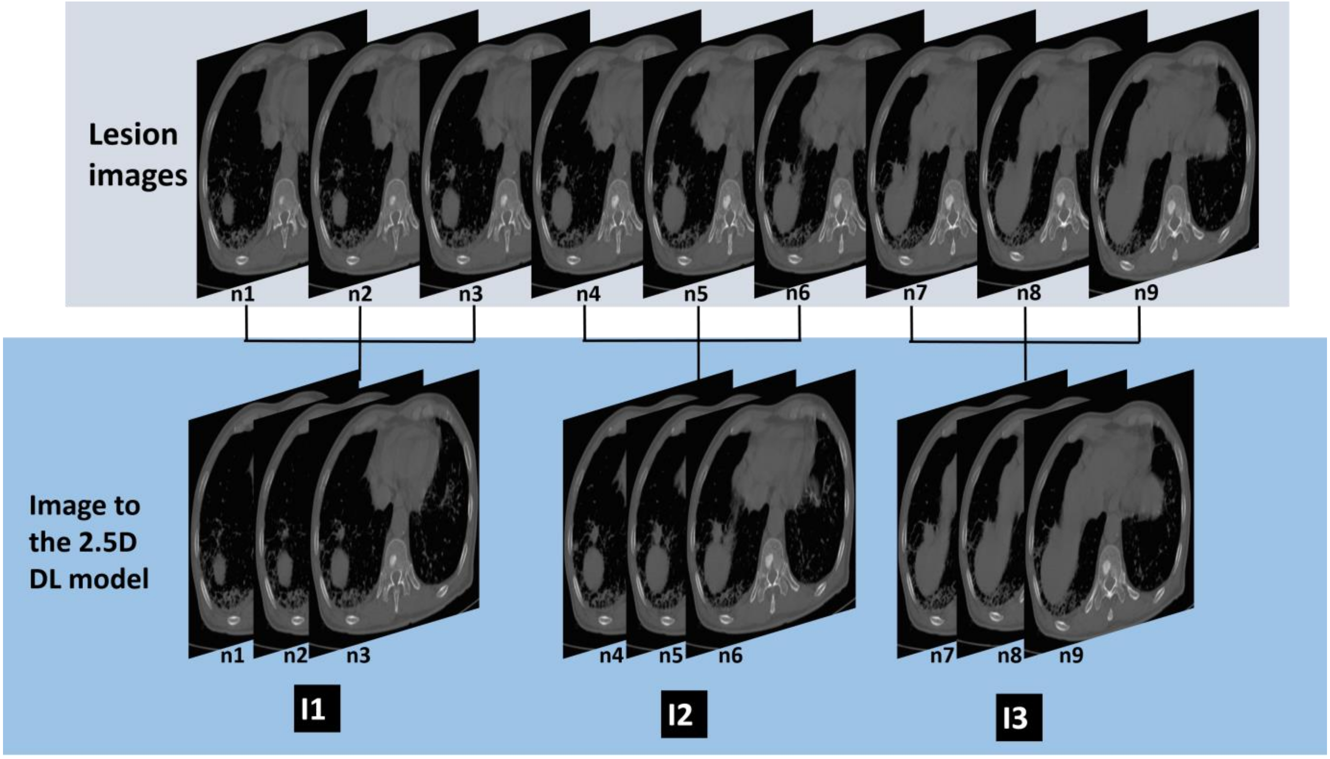

FIG S1: The process of generating and verifying images input to the 2.5 DL model. Three adjacent image slices were combined to create a three-channel image input. Labels n1 to n9 represent the slice numbers of the axial CT images, and I1 to I3 denote the three-layer images input to the 2.5 DL model
